# Reperfusion Arrhythmias Increase after Superior Cervical Ganglionectomy Due to Conduction Disorders and Changes in Repolarization

**DOI:** 10.3390/ijms21051804

**Published:** 2020-03-06

**Authors:** Natalia Jorgelina Prado, Estela Maris Muñoz, Luz Estefanía Farias Altamirano, Francisco Aguiar, Amira Zulma Ponce Zumino, Francisco Javier Sánchez, Roberto Miguel Miatello, Esther Pueyo, Emiliano Raúl Diez

**Affiliations:** 1 Facultad de Ciencias Médicas, Universidad Nacional de Cuyo, Mendoza 5500, Argentina; nprado@mendoza-conicet.gob.ar (N.J.P.); franciscoaguiar_7@hotmail.com (F.A.); aponce@fcm.uncu.edu.ar (A.Z.P.Z.); franchosanchez@gmail.com (F.J.S.); rmiatell@fcm.uncu.edu.ar (R.M.M.); 2 Institute of Medical and Experimental Biology of Cuyo, IMBECU-UNCuyo-CONICET, Mendoza 5500, Argentina; 3 Laboratory of Neurobiology: Chronobiology Section, Institute of Histology and Embryology of Mendoza, IHEM-UNCUYO-CONICET, Mendoza 5500, Argentina; munoz.estela@fcm.uncu.edu.ar (E.M.M.); luzver86@gmail.com (L.E.F.A.); 4 BSICOS Group, I3A, IIS Aragón, University of Zaragoza, 50018 Zaragoza, Spain; 5 CIBER-BBN, 28029 Madrid, Spain

**Keywords:** melatonin, superior cervical ganglia, arrhythmia, melatonin receptors, K_ATP_ channels, connexin 43

## Abstract

Pharmacological concentrations of melatonin reduce reperfusion arrhythmias, but less is known about the antiarrhythmic protection of the physiological circadian rhythm of melatonin. Bilateral surgical removal of the superior cervical ganglia irreversibly suppresses melatonin rhythmicity. This study aimed to analyze the cardiac electrophysiological effects of the loss of melatonin circadian oscillation and the role played by myocardial melatonin membrane receptors, SERCA_2A_, TNFα, nitrotyrosine, TGFβ, K_ATP_ channels, and connexin 43. Three weeks after bilateral removal of the superior cervical ganglia or sham surgery, the hearts were isolated and submitted to ten minutes of regional ischemia followed by ten minutes of reperfusion. Arrhythmias, mainly ventricular tachycardia, increased during reperfusion in the ganglionectomy group. These hearts also suffered an epicardial electrical activation delay that increased during ischemia, action potential alternants, triggered activity, and dispersion of action potential duration. Hearts from ganglionectomized rats showed a reduction of the cardioprotective MT_2_ receptors, the MT_1_ receptors, and SERCA_2A_. Markers of nitroxidative stress (nitrotyrosine), inflammation (TNFα), and fibrosis (TGFβ and vimentin) did not change between groups. Connexin 43 lateralization and the pore-forming subunit (Kir6.1) of K_ATP_ channels increased in the experimental group. We conclude that the loss of the circadian rhythm of melatonin predisposes the heart to suffer cardiac arrhythmias, mainly ventricular tachycardia, due to conduction disorders and changes in repolarization.

## 1. Introduction

Sudden death is the leading cause of cardiovascular mortality worldwide [1]. Sudden cardiac death varies depending on the prevalence of coronary heart disease, and it has a circadian distribution with a peak in the morning hours [2,3,4,5,6,7,8]. Preservation of circadian oscillations from the level of intracellular organelles to the integrated cardiovascular system appears to be essential for maintaining health and preventing diseases [8,9,10,11,12].

The pineal gland is the main source of the circulating nocturnal melatonin, which impacts the whole physiology and presents cardioprotective properties [13,14]. The administration of high pharmacological doses of melatonin protects against oxidative and nitroxidative stress, arrhythmias, and inflammation, reduces blood pressure, and attenuates metabolic syndrome [15,16,17]. A cardioprotective role of physiological levels of melatonin has been observed in the clinical scenario, and it has been tested under experimental conditions using pinealectomy [3,5,6,13,14,18]. 

The release of melatonin oscillates following a light–dark cycle through a multisynaptic pathway between the eyes and the pineal gland [19,20]. Light has an inhibitory effect on melatonin secretion along a path that begins in the retina and modulates the activity of the suprachiasmatic nuclei (SCN), where the master circadian clock is located [8]. The SCN send efferents that will impact indirectly in the superior cervical ganglia (SCG), from which sympathetic nerve fibers emerge towards the pineal gland. 

We recently reported the metabolic effects of chronic bilateral superior cervical ganglionectomy (SCGx) in rats [17,19]. This surgical model results in a functional pinealectomy. In fact, bilateral SCGx is a reliable procedure to prevent the nocturnal activation of the enzyme arylalkylamine-N-acetyltransferase, irreversibly suppressing melatonin increase during dark hours [17,19].

Melatonin receptors play a significant role in the cardioprotective actions of melatonin [7,21,22,23,24,25,26,27,28,29,30,31,32]. MT_2_ knockout mice lost the cardioprotective actions of melatonin against ischemia/reperfusion injury [24]. Luzindole, a melatonin receptor antagonist, abrogates the antiarrhythmic effects of melatonin under hypokalemic proarrhythmic conditions [23]. Acute melatonin receptor activation shortened action potential duration (APD) at 50% of repolarization, but it did not change the duration at 90% of repolarization or QT interval duration in the ECG during hypokalemia. Anti-adrenergic actions of melatonin are also blocked by luzindole [21]. Melatonin also reduces calcium overload by inhibition of inositol trisphosphate receptor expression and promotion of sarco/endoplasmic reticulum Ca^2+^-ATPase 2A (SERCA_2A_) expression via the extracellular-signal-regulated kinase 1 pathway in cardiomyocytes [33]. ATP-regulated potassium channels (K_ATP_) are involved in ischemia/reperfusion arrhythmias, and recently, a modulatory effect of melatonin was described for these channels [34,35,36]. Connexins contribute to electrical coupling and signal propagation across myocardial tissue. We recently showed that melatonin preserved myocardial connexin 43 distribution and phosphorylation in a receptor-dependent manner [23]. Chronic melatonin administration preserves connexin topology and functionality, making the heart more resistant to arrhythmic stimuli [37]. Chronic melatonin administration at pharmacological levels prevents pro-arrhythmic myocardial remodeling induced by kidney disease [38,39]. However, the role of melatonin circadian oscillation in these critical proteins involved in arrhythmogenesis is scarce.

This study aimed to analyze the cardiac electrophysiological effects of the loss of melatonin circadian oscillation, and the role played by myocardial melatonin membrane receptors, SERCA_2A_, TNFα, nitrotyrosine, TGFβ, KATP channels, and connexin 43.

## 2. Results

### 2.1. Electrophysiological Effects of SCGx in Isolated Rat Hearts Submitted to Regional Ischemia/Reperfusion

**Figure 1:**
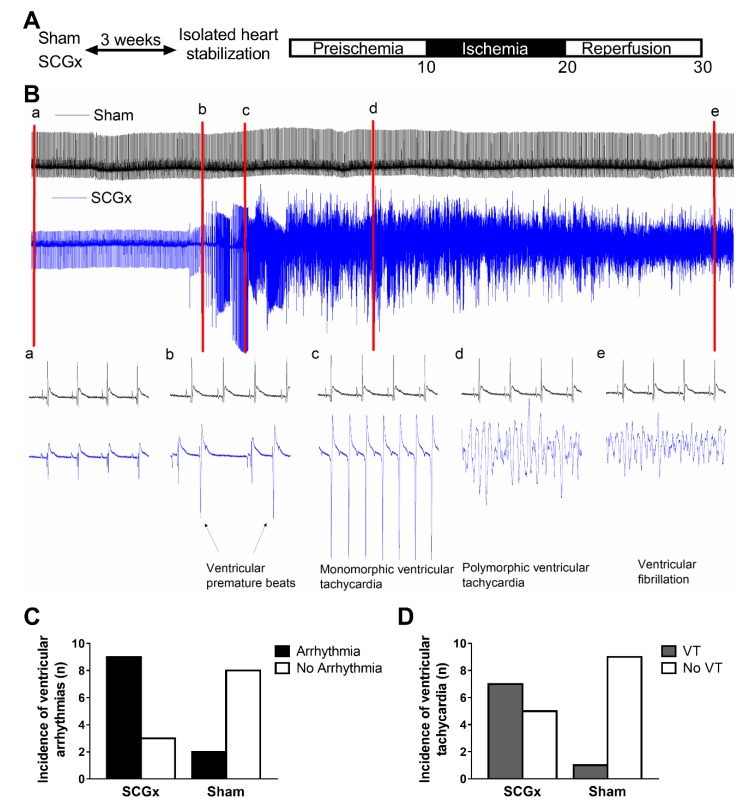
Hearts from SCGx rats suffered severe reperfusion arrhythmias. (**A**) Experimental protocol: three weeks after bilateral superior cervical ganglionectomy (SCGx) or sham operation, hearts were isolated and perfused according to the Langendorff technique. After stabilization, electrocardiograms (ECG) and action potentials were recorded during 10 min of preischemia, 10 min of regional myocardial ischemia, and 10 min of reperfusion. (**B**) Representatives ECG from the first 3 min of reperfusion. The red marks and their corresponding lowercase letters indicate where the time scale was extended to 1 s, and they display the different types of arrhythmias identified. (**C**) The ganglionectomized animals had a higher number of arrhythmias compared to those sham-operated (*p* = 0.03 by Fisher’s exact test). (**D**) Ventricular tachycardia increased in the SCGx group (*p* = 0.031 by Fisher’s exact test).

Reperfusion ventricular arrhythmias increased in rat hearts isolated after three weeks of surgical removal of the superior cervical ganglia (SCGx) (Figure 1). Ventricular premature beats and ventricular tachycardia were the main types of arrhythmias seen in the SCGx group, while ventricular fibrillation did not increase after surgeries (2/12 in SCGx and 0/10 in sham). Ventricular tachycardia also lasted longer in SCGx (median 36 s, IQR 0-66) than in sham hearts (median 0, IQR 0-0, *p* = 0.0093 by Mann–Whitney test).

**Figure 2:**
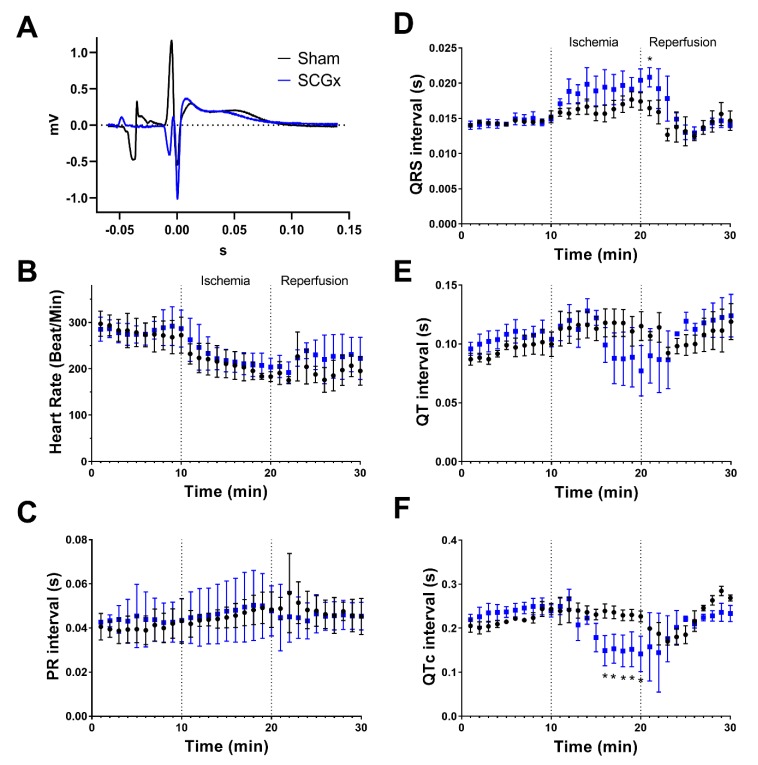
Heart rate and ECG intervals during ischemia/reperfusion. (**A**) Representative ECG from the preischemic period. PR, QRS, or QT intervals did not differ between groups. (**B**) Heart rate decreased during ischemia and partially recovered during reperfusion in both groups. (**C**) PR interval showed more dispersion during ischemia in the SCGx group. (**D**) QRS interval increased during ischemia and reached a significant difference at the first minute of reperfusion in the SCGx animals. (**E**) QT interval showed more dispersion during the last minutes of ischemia and the beginning of reperfusion in the SCGx group. (**F**) QTc from SCGx hearts shortened during the last five minutes of ischemia.

During preischemia, heart rate and ECG intervals (PR, QRS, QT, QTc) did not show differences among groups (Figure 2). Ischemia induced progressive bradycardia in both groups. PR interval dispersed more in SCGx than in sham hearts during ischemia. QRS increased during ischemia and reached a significant difference at the beginning of reperfusion in the experimental group. QT interval shortened during the last 5 min of ischemia in SCGx hearts, and this shortening was clearer when QT intervals were corrected by the heart rate (QTc). 

**Figure 3:**
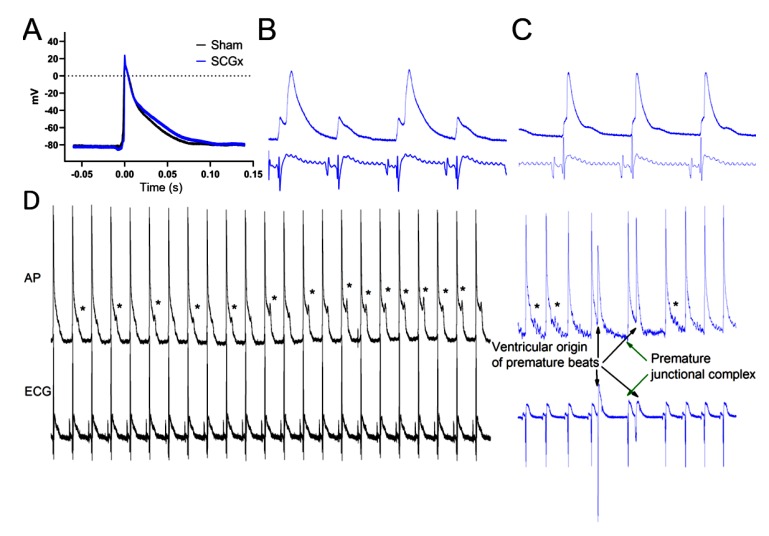
Epicardial action potential. (**A**) Averaged action potentials from preischemic recordings display a lengthening of 50% of repolarization in cardiomyocytes from SCGx hearts. (**B**) During ischemia, action potential (upper trace) activation delayed in respect of the beginning of the QRS complex of the ECG (lower trace). This is an indicator of reduced local propagation and suffered conduction blockage (2:1) causing repolarization alternants (blue traces correspond to 1 s). (**C**) Local activation delay persisted, and action potential shortened during the last minutes of ischemia, and early after-depolarization frequently occurred in SCGx group (blue traces correspond to 1 s). (**D**) During reperfusion, hearts from the sham group displayed early after-depolarizations (indicated by the asterisks), but they did not trigger arrhythmias as it can be seen in the ECG (black traces correspond to 6 s). In hearts from the SCGx group, early after depolarization triggered premature beats. Additionally, junctional complexes were also detected. The absence of P wave and QRS morphology like supraventricular activation (green arrows) are assumed to be an escape beat from the atrioventricular conduction system.

Epicardial action potential duration (APD) was longer in SCGx hearts during preischemia, but this prolongation did not modify the QT interval duration in the ECG (Figure 2 and Figure 3A). The prolongation in APD was significant from 50% of repolarization (Figure 3A). 

Ganglionectomy induced a delay in action potential upstroke respective to the onset of the QRS complex during ischemia compared with the sham group (22.1 ± 1.3 ms vs. 12.5 ± 1.2 ms, *p* < 0.05 by repeated measures ANOVA) (Figure 3B). In addition, SCGx hearts suffered a more pronounced action potential shortening during the last three minutes of ischemia (Figure 3C).

Early after-depolarizations triggered premature ventricular beats only in the SCGx group. Other types of ectopic activity, like premature junctional complex, occurred in hearts from ganglionectomized rats (Figure 3D).

### 2.2. SCGx Reduced the Expression of Myocardial Melatonin Receptors and SERCA_2A_

**Figure 4:**
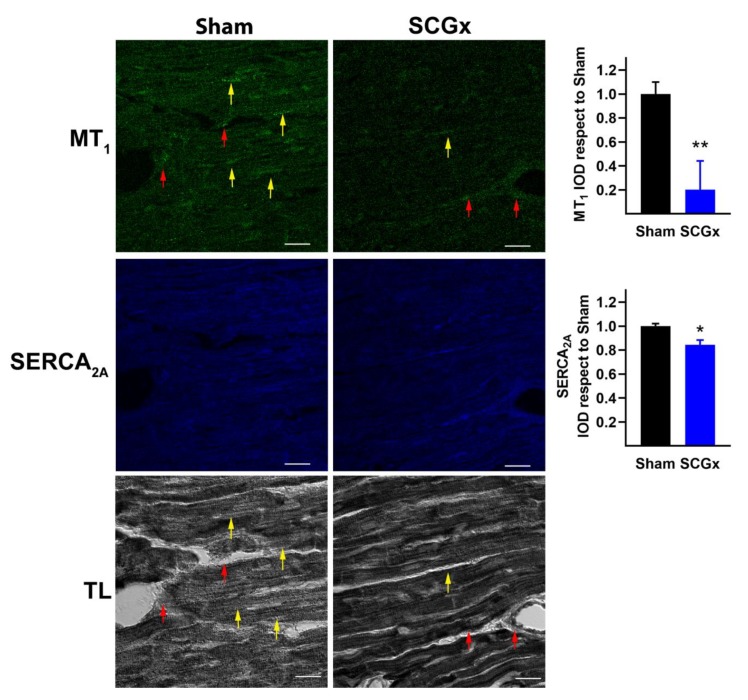
Melatonin receptor type 1 (MT_1_) and SERCA_2A_ in myocardial tissue. Confocal immunofluorescence microscopy showed that MT_1_ and SERCA_2A_ decreased in cardiomyocytes from the SCGx group (yellow arrows). Vascular expression of MT_1_ receptors remained unchanged (red arrows). Transmitted light (TL) images showed the vascular and myocytic location of MT_1_ receptors. Integrated optical density (IOD) was quantified as the area (>3 pixels connected) multiplied by the average intensity. Bars in the right-hand column indicate mean and SEM values relative to the level measured in the sham group. The white scale bars in the lower right corners indicate 20 µM. In both groups, a total of 18 pictures were analyzed, six from three different hearts. * *p* < 0.05 and ** *p* < 0.01 vs. Sham by unpaired t-test with Welch’s correction.

**Figure 5:**
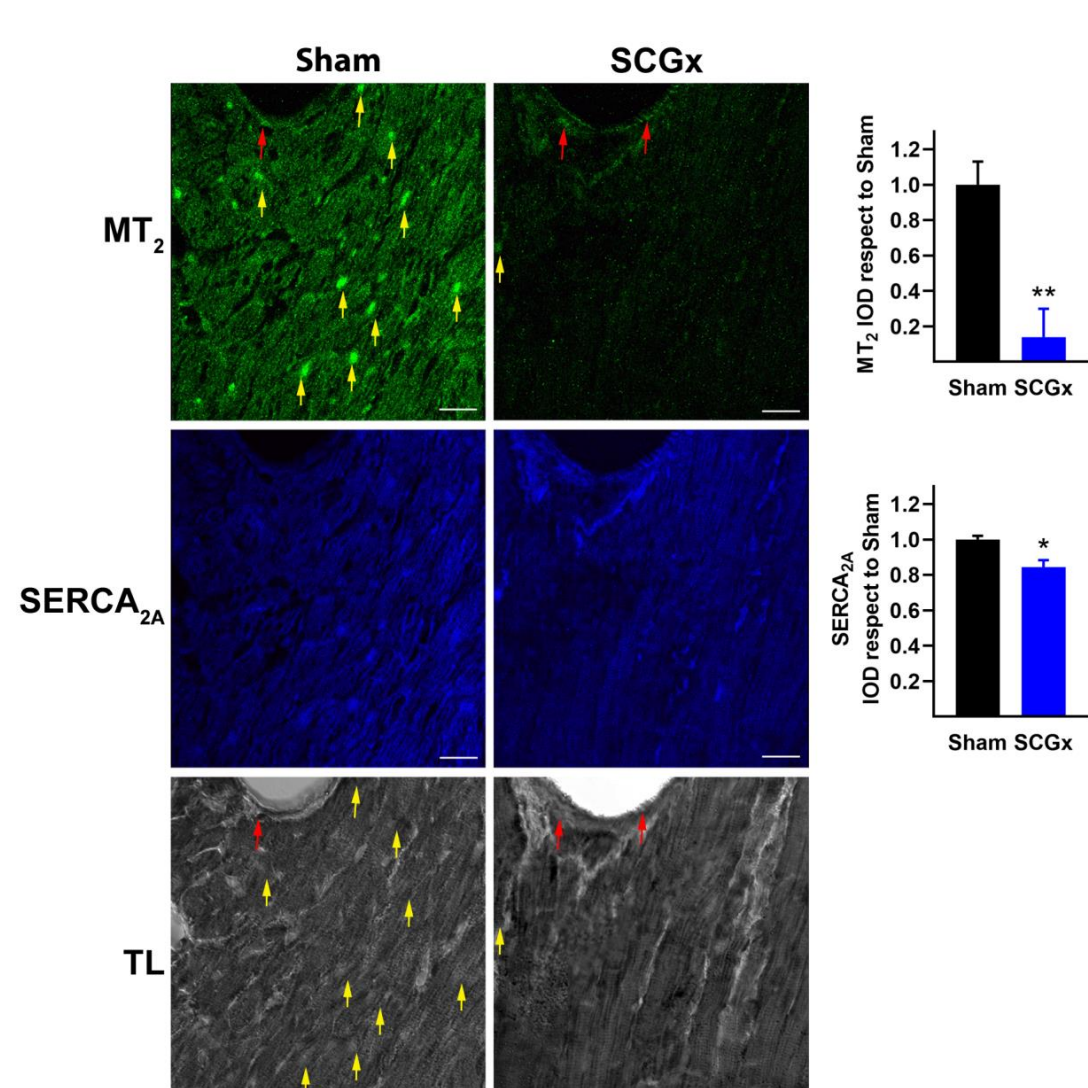
Melatonin receptor type 2 (MT_2_) and SERCA_2A_ in myocardial tissue. The cardioprotective MT_2_ receptors decreased in myocytes from the SCGx group (yellow arrows). MT_2_ receptors persisted in vascular tissue (red arrows). The middle panel is shown to confirm the reduction of SERCA_2A_ in the SCGx hearts. Transmitted light (TL) images revealed the vascular and myocytic location of MT_2_ receptors. Bars in the right-hand column indicate the mean integrated optical density (IOD) and SEM values relative to the level measured in the Sham group. The white scale bars in the lower right corners indicate 20 µM. In both groups, a total of 18 pictures were analyzed, six from three different hearts. * *p* < 0.05 and ** *p* < 0.01 vs. Sham by unpaired t-test with Welch’s correction.

The expression of both melatonin receptors, MT_1_ and MT_2_, decreased in cardiomyocytes of hearts from the SCGx group (Figure 4 and Figure 5). However, the vascular expression of both receptors remained unchanged (red arrows in Figure 4 and Figure 5). In addition, the levels of SERCA_2A_ declined in myocytes of ganglionectomized rats. 

### 2.3. SCGx Increased K_ATP_ Channels and Connexin 43 Lateralization, Without Changing TNFα or Nitrotyrosine

**Figure 6:**
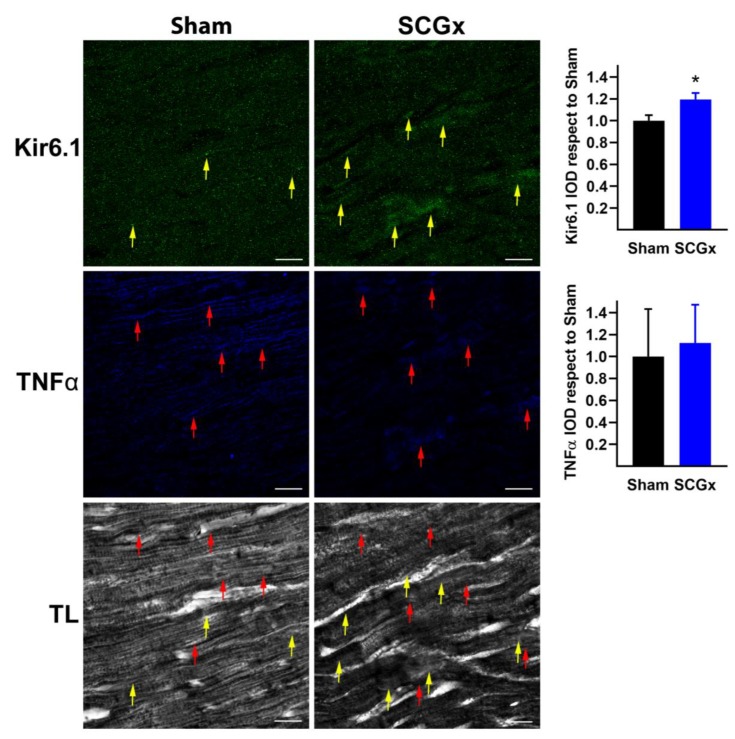
The pore-forming subunit (Kir6.1) of K_ATP_ and tumor necrosis factor-alpha (TNFα) in SCGx rats. Kir6.1 increased in cardiomyocytes from ganglionectomized rats (yellow arrows). The levels of the inflammatory marker TNFα were low in ventricular samples of both groups (red arrows). Bars in the right-hand column indicate the mean integrated optical density (IOD) and SEM values relative to the level measured in the Sham group. The white scale bars in the lower right corners indicate 20 µM. In both groups, a total of 18 pictures were analyzed, six from three different hearts. * *p* < 0.05 vs. Sham by unpaired t-test with Welch’s correction.

The pore-forming subunit (Kir6.1) of ATP-regulated potassium channels increased in the SCGx hearts (Figure 6). This result is consistent with the action potential and QTc shortening shown in Figure 2 and Figure 3. ATP depletion and ADP increase during ischemia; furthermore, oxidative stress and acidosis are stimuli for the sulfonylurea receptor subunits that open Kir6.1.

Tumor necrosis factor α (TNFα), a pro-inflammatory and pro-arrhythmic stimulus, did not increase after bilateral SCGx (Figure 6). Indeed, the levels found in myocardial tissue were low in both groups.

**Figure 7:**
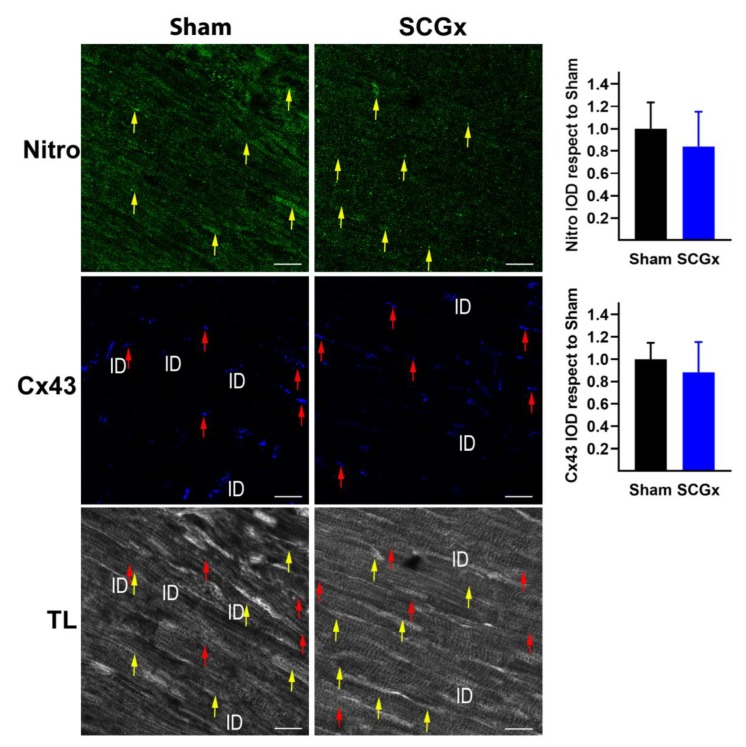
Myocardial nitroxidative stress and connexin 43 (Cx43) after bilateral SCGx. Nitrotyrosine (Nitro) did not change between groups (yellow arrows). Sham hearts showed Cx43 mainly at the intercalated discs (ID), and a scarce signal was also observed at the lateral levels of myocytes (red arrows). SCGx reduced Cx43 at ID, and most of the signal was located at the lateral borders of the myocytes. Bars in the right-hand column indicate the mean integrated optical density (IOD) and SEM values relative to the level measured in the Sham group. The white scale bars in the lower right corners indicate 20 µM. In both groups, a total of 18 pictures were analyzed, six from three different hearts.

Nitrotyrosine is a relatively stable marker of nitroxidative stress that is formed by peroxynitrite interaction with tyrosine. Like TNFα, myocardial nitrotyrosine was not affected by chronic SCGx (Figure 7). 

Electrical signals propagate between cardiomyocytes through channels formed by connexin 43 (Cx43), in areas of the cells called intercalated discs (ID). Hearts from the Sham group showed abundant levels of Cx43 at ID and fewer signals in the lateral borders of the cardiomyocytes (see red arrows in Figure 7). The bilateral SCGx reduced the levels of Cx43 at the ID and increased the lateral levels. The total intensity of Cx43 did not change between groups, indicating a lateralization. 

### 2.4. SCGx Did Not Increase Markers of Fibrosis

**Figure 8:**
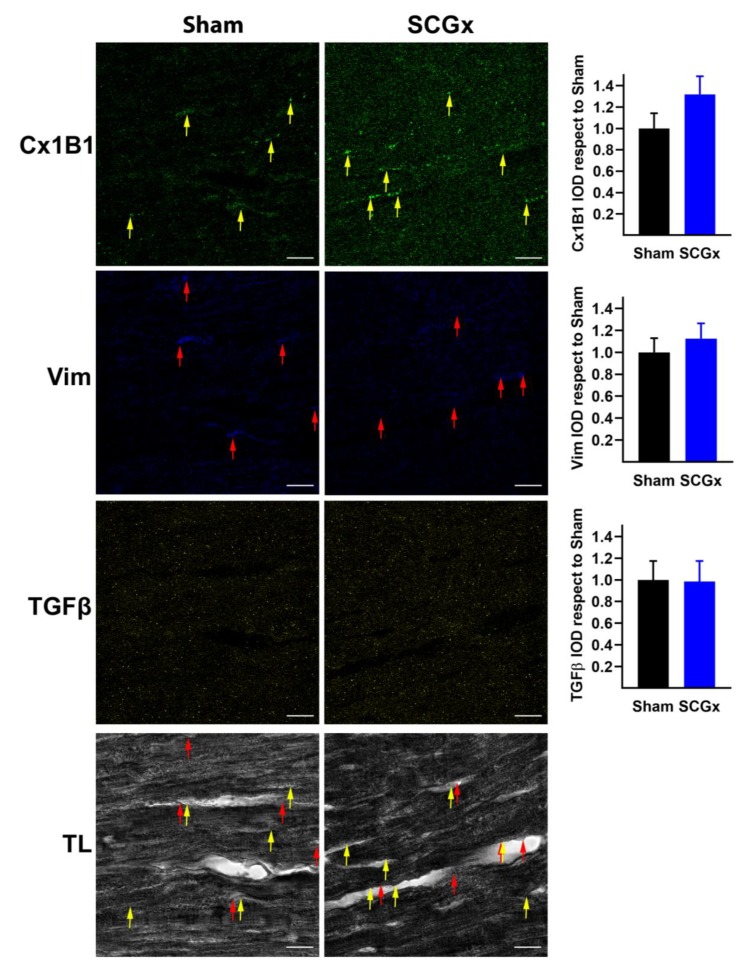
SCGx did not increase fibrosis. The unphosphorylated connexin 43 detected by the Cx1B1 antibody was lateralized mainly to the vascular side of the myocytes (yellow arrows), close to fibroblast marker vimentin (Vim, red arrows). Sham and SCGx groups showed the same levels of Vim and the profibrotic stimulus TGFβ (yellow staining). Bars indicate the mean integrated optical density (IOD) and SEM values relative to the level measured in the Sham group. The white scale bars in the lower right corners indicate 20 µM. In both groups, a total of 18 pictures were analyzed, six from three different hearts.

The fibrotic stimulant TGBβ and the fibroblast marker vimentin remained unchanged in both sham and SCGx groups (Figure 8). The unphosphorylated connexin 43 at serine 368 that is identified by the monoclonal antibody Cx1B1 displayed a perivascular distribution (see yellow arrows in Figure 8) near to Vim (red arrows) in both groups.

## 3. Discussion

Physiological circadian oscillation of melatonin protects the heart from reperfusion arrhythmias. Previous studies showed that pinealectomy increased myocardial injury and arrhythmias [6,13,14,40]. Our results confirm the physiological importance of rhythmic melatonin secretion, without removing the pineal gland or using any antiadrenergic agent. Although bilateral superior cervical ganglionectomy (SCGx) is a surgical procedure, this technique is less invasive than pinealectomy [17,19]. On the other hand, SCGx is more selective than a pharmacological adrenergic blockade. In addition to technical considerations, electrophysiological and molecular mechanisms of arrhythmias were investigated in young adult Wistar rats subjected to chronic SCGx or sham surgery.

The combination of triggered activity and variability in ventricular activation and repolarization is an appropriate substrate for the initiation and maintenance of re-entry circuits [41,42,43]. Epicardial action potentials revealed higher vulnerability to arrhythmic triggers in hearts from SCGx rats (Figure 3D). Bilateral ganglionectomy impaired ventricular activation of cardiomyocytes (Figure 3B,C) and also at the whole heart level, as evidenced in the QRS lengthening during ischemia and reperfusion (Figure 2D). Finally, the dispersion of QT intervals and QTc shortening during ischemia facilitate functional re-entrance, as an additional mechanism to explain the increase in ventricular tachycardia observed in the SCGx group (Figure 1, Figure 2 and Figure 3).

Triggered activity is strongly related to calcium overload, and it can be attributed to reduced levels of SERCA_2A_ in our experimental model [33,44]. SERCA_2A_ is the main ATPase responsible for calcium reuptake at the sarcoplasmic reticulum [45,46]. Other homeostatic mechanisms regulate calcium, but the SERCA_2A_ reduction described here could be a strong indicator of calcium-triggered events during ischemia/reperfusion. 

The APD prolongation could be related to the loss of melatonin receptors at cardiomyocyte membranes. Melatonin receptors are coupled to Kir3.x channels, and melatonin receptor activation shortens the action potential duration [23,47]. However, we recently showed that melatonin-induced changes on myocardial repolarization were associated with melatonin antioxidative properties but not with its antiarrhythmic actions [15,48,49]. Our negative results with nitrotyrosine (Figure 7) as a marker of nitroxidative stress agree with antioxidant been unrelated to antiarrhythmic effects. On the other side, the increase in the Kir6.1 subunit observed in SCGx hearts (Figure 6) could shorten action potential under stressful conditions like ischemia, and it could predispose the hearts to re-entrance circuits. 

The proarrhythmic effects of SCGx might be related to altered activation and connexin 43 redistribution. Both mechanisms are potential explanations of sustained reperfusion tachycardia. Conduction disturbances in SCGx hearts could be related to the redistribution of connexin 43, and the lateralization of unphosphorylated forms of the Cx43 reported here and concurred with previous studies [23]. Our results are also relevant for other diseases with increased arrhythmic risk and chronodisruption like coronary artery disease, non-dipping hypertension, sleep apnea, obesity, and chronic kidney disease [5,10,16]. Especially, connexin 43 lateralization can aggravate pathologies with diminished conduction velocity due to fibrosis or genetic mutations [37,39,50].

The negative results described here for TNFα and markers of fibrosis are interesting. TNFα is an inflammatory and proarrhythmic factor [51,52,53]. Our results indicate that myocardial inflammation is not a key arrhythmogenic factor in this model because TNFα remained unchanged in ganglionectomized hearts. The profibrotic TGBβ and the fibroblast marker vimentin did not increase after SCGx, indicating a functional rather than a structural substrate for the SCGx-induced arrhythmias [15,38,39].

Future research using the SCGx model with or without administration of exogenous melatonin will help to further understand the potential role of melatonin as an endogenous cardioprotector that keeps pace with physiological challenges.

## 4. Materials and Methods

The IAUC of the Faculty of Medicine, National University of Cuyo (Protocol Nº 74/2016-2019) approved the present study and monitored that experiments were conducted in accordance with the National Institutes of Health’s Guide for Care and Use of Laboratory Animals and the Animal Research: Reporting In Vivo Experiments (ARRIVE) Guidelines. 

### 4.1. Animals

Male Wistar rats were raised until 3 months of age in our colony under a 12 h–12 h light–dark cycle (with Zeitgeber time 12 (ZT 12) defined as the time of lights off; light intensity averaging 300 lux at the cage level), in a controlled environment with food and water ad libitum.

### 4.2. Surgery

Bilateral superior cervical ganglionectomy (SCGx) was performed as previously described [17,19,20,54]. Briefly, under ketamine/xylazine anesthesia (50 mg/kg of body weight and 5 mg/kg of body weight, respectively), the ventral neck region was shaved and disinfected. A 2.5 cm vertical incision exposed the salivary glands. After gentle retraction of the glands, the underlying muscles were uncovered. The carotid triangles served as a reference for SCG identification and removal after sectioning the sympathetic trunks, the internal carotid nerves, and the external carotid nerves. For sham-operated animals, the same procedure was performed but the ganglia were not touched nor removed. SCGx and sham rats were kept in the animal facility for three weeks until their sacrifice around ZT6 (middle of light phase); ZT when the circulating melatonin levels are expected to be minimal [55]. 

### 4.3. Electrophysiological Studies

#### 4.3.1. Arrhythmias

Ventricular arrhythmias in isolated rat hearts were classified according to the Lambeth Convention [56]. Ischemia/reperfusion experiments were performed at ZT 6 ± 1 h. 

#### 4.3.2. Electrocardiograms and Action Potentials

Cardiac electrograms and transmembrane action potentials from left ventricle epicardial cells were obtained from isolated rat hearts, as previously described [15,23].

### 4.4. Confocal Immunofluorescence Microscopy

Hearts for immunostaining were fixed in 4% paraformaldehyde in phosphate-buffered saline at 4 °C at the end of the experimental protocol. All the immunohistochemical procedures were performed as previously described [39]. Sections were stained with the following primary antisera: rabbit polyclonal anti-MTNR1A (MT_1_, Origen, TA321735, dilution 1:500), rabbit polyclonal anti-MTNR1B (MT_2_, Origen, AP01322PU-N, dilution 1:500), rabbit polyclonal anti-Cx43 (Cx43, Abcam, ab11370, dilution 1:1000), rabbit polyclonal anti-Kir6.1 (Kir6.1, Thermofisher, PA5-48354, dilution 1:500); mouse monoclonal anti-SERCA_2A_ (SERCA_2A_, Abcam, ab2817, dilution 1:500), mouse monoclonal anti-unphosphorylated connexin 43 at serine 368 (CX-1B1, Thermofisher, #13-8300, dilution 1:200); mouse monoclonal anti-vimentin (Vimentin, Sigma, V6630 dilution 1:200); mouse monoclonal anti-nitrotyrosine (3-nitroityrosine, Santa Cruz, sc-32757, dilution 1:200); and mouse monoclonal anti-TGFβ (TGFβ, Santa Cruz, 3C11). The secondary antisera included anti-rabbit conjugated with Alexa Fluor 488 and anti-mouse labelled with the Alexa Fluor 405 fluorophore (Jackson ImmunoResearch Laboratories Inc., West Grove, PA, USA, dilution 1:500). Images were obtained with a Confocal Zeiss LSM 880 and processed with Zen Blue 2.5 software (Carl Zeiss Microscopy GmbH, 2018). The maximum intensity of 30 to 50 z stacks were projected and used for the quantitative analysis. Integrated optical density (IOD) was quantified as the area (>3 pixels connected) multiplied by the average intensity using ImageProPlus version 4.5, 2001 for windows (Media Cybernetics, Inc., Rockville, MD, USA). Values of IOD are expressed as mean and SEM relative to the level measured in the Sham group. For all the images from both groups, a total of 18 pictures per immunofluorescence channel were analyzed, six from three different hearts. 

### 4.5. Statistical Analysis

Data were expressed as mean ± SEM. Inferential analysis was performed using ANOVA or repeated-measures ANOVA, followed by Bonferroni post-test and contingency tables, which were treated by Fisher exact test, as appropriate. The duration of reperfusion arrhythmias was expressed as the median and interquartile range (IQR) and analyzed using the Mann–Whitney U test. Immunofluorescence images were compared by unpaired t-test with Welch’s correction. The GraphPad Prism version 8.4.0, 2020 for Windows (GraphPad Software, San Diego, CA, USA, www.graphpad.com) was used for the statistical analysis.

## Abbreviations

ADP	action potential duration
Cx43	connexin 43
IOD	integrated optical density
MT_1_	melatonin type 1 receptor
MT_2_	melatonin type 2 receptor
Nitro	nitrotyrosine
SCGx	superior cervical ganglionectomy
SERCA_2A_	sarco/endoplasmic reticulum Ca^2+^-ATPase, paralog 2A
TGFβ	transforming growing factor β
TL	transmitted light
TNFα	tumor necrosis factor α
